# Human recombinant activated protein C-coated stent for the prevention of restenosis in porcine coronary arteries

**DOI:** 10.1007/s10856-015-5580-6

**Published:** 2015-09-28

**Authors:** Dominika Lukovic, Noemi Nyolczas, Rayyan Hemetsberger, Imre J. Pavo, Aniko Pósa, Boris Behnisch, Gerhard Horak, Katrin Zlabinger, Mariann Gyöngyösi

**Affiliations:** Department of Cardiology, Medical University of Vienna, Währinger Gürtel 18-20, 1090 Vienna, Austria; Translumina GmbH, Hechingen, Germany; amacord GmbH Vienna, Vienna, Austria

## Abstract

Activated protein C (APC), an endogenous protein, inhibits inflammation and thrombosis and interrupts the coagulation cascade. Here, we investigated the effect of human recombinant APC on the development of neointimal hyperplasia in porcine coronary arteries. Yukon Choice bare metal stents were coated with 2.6 µg APC/mm^2^. Under general anesthesia, APC-coated and bare stents were implanted in the left anterior descending and circumflex coronary arteries of 10 domestic pigs. During the 4-week follow-up, animals were treated with dual antiplatelet therapy and neointimal hyperplasia was evaluated via histology. Scanning electron microscopy indicated successful but unequal coating of stents with APC; nearly complete drug release occurred within 4 h. Enzyme-linked immunosorbent assay revealed that intracoronary stent implantation rapidly increased the levels of monocyte chemoattractant protein-1, an effect that was inhibited by APC release from the coated stent. Fibrin deposition and adventitial inflammation were significantly decreased 1 month after implanting APC-coated stents versus bare stents, paralleled by significantly smaller neointimal area (0.98 ± 0.92 vs. 1.44 ± 0.91 mm^2^, *P* = 0.028), higher lumen area (3.47 ± 0.94 vs. 3.06 ± 0.91 mm^2^, *P* = 0.046), and lower stenosis area (22.2 ± 21.2 % vs. 32.1 ± 20.1 %, *P* = 0.034). Endothelialization was complete with APC-coated but not bare (90 %) stents. P-selectin immunostaining revealed significantly fewer activated endothelial cells in the neointima in the APC group (4.6 ± 1.9 vs. 11.6 ± 4.1 %, *P* < 0.001). Thus, short exposure of coronary arteries to APC reduced inflammatory responses, neointimal proliferation, and in-stent restenosis, offering a promising therapy to improve clinical outcomes of coronary stenting. However, coating stents with APC for prolonged, controlled drug release remains technically challenging.

## Introduction

Coated stents have been shown to overcome the high in-stent restenosis rate of bare metal stents (BMSs). Several candidate drugs for stent coatings were tested: molecules in the limus group (e.g. sirolimus, everolimus, tacrolimus, biolimus), molecules in the taxol group (e.g. taxane, paclitaxel), estradiol, statin, glitazon, and combinations of substances (e.g. rapamycin with estradiol) [1]. However, most coating materials (e.g. cytostatics, antiproliferative drugs, or the drug-carrying polymer itself) may exert toxic effects on the arterial wall, causing vessel aneurysms and focal necrosis with accompanying severe inflammatory reactions [2].

Activated protein C (APC) is an endogenous protein that inhibits inflammation, apoptosis, and thrombosis, promotes fibrinolysis, and is an important modulator of the coagulation and inflammation systems [3–5]. APC is converted from its inactive precursor, protein C, by thrombin coupled to thrombomodulin [5]. Importantly, APC acts against local cell proliferation and thrombosis, factors known to contribute to the progression of coronary artery disease and the development of restenosis after percutaneous coronary intervention or stent thrombosis via its anti-inflammatory, antithrombotic, fibrinolytic, and antiapoptotic functions.

APC inhibits monocyte production of several inflammatory cytokines (tumor necrosis factor alpha and interleukins 1, 6, and 8) and limits the migration of monocytes and neutrophils to injured endothelium by binding selectins [4, 5]. APC also interrupts the coagulation cascade by inhibiting the generation of thrombin via inactivating factors Va and VIIIa [3, 5] and by reducing intravascular fibrin accumulation [5–10]. Further, APC promotes fibrinolysis by attenuating the production of thrombin, thereby limiting the activation of thrombin-activated fibrinolysis inhibitor [8], and increases the fibrinolytic response indirectly by inhibiting plasminogen activator inhibitor-1 [11]. These factors also play a role in local acute and chronic vascular inflammation and thrombosis after percutaneous coronary intervention.

Based on these anti-inflammatory, antithrombotic, and fibrinolytic properties, APC may constitute an optimal stent-coating substance for the prevention of in-stent restenosis and stent thrombosis. Initial clinical results indicated that human recombinant APC (hrAPC, drotrecogin alfa (Xigris), Eli Lilly, Indianapolis, IN, USA) reduced mortality in sepsis [12–14], including in patients with severe sepsis and septic shock. However, Xigris was withdrawn from the market (http://www.fda.gov/Drugs/DrugSafety/ucm277114.htm) after large randomized studies failed to detect survival benefits.

Based on the expected antithrombotic effect of APC, rabbit iliac arteries were treated with APC-loaded stents; this treatment inhibited platelet deposition on stent wires, with indirect evidence of APC elution [15]. In the current investigation, we sought to explore the potential benefits of hrAPC in a porcine pre-clinical stenting model, to investigate the safety and efficacy of hrAPC-coated stents, and to determine the effects of hrAPC-coated stents on the development of neointimal hyperplasia, local endothelialization, and the inhibition of endothelial-cell activation. We chose Xigris as the coating substance because additional components of this drug could inhibit the immediate proteolytic inactivation of APC and promote the retention of hrAPC on the stent surface.

## Materials and methods

### Coating of stents with hrAPC

Sixteen Yukon Choice BMSs (Translumina GmbH, Hechingen, Germany) with surface micropores that allowed polymer-free drug loading were coated with solution containing 300 µg hrAPC in sterile water (as recommended by the manufacturer) using the Translumina Stent Coating System (T-SCM 2003, Translumina). After coating, the stent was left in the cartridge to ensure attachment of the drug to the stent surface and to allow the coating to dry. All necessary care was taken to maintain sterility.

### In vitro drug-release kinetics

After coating, five stents were removed from the sterile plastic bag, dilated with the stent balloon, and cut from the catheter. Dilated hrAPC-coated stents were placed in an artificial vessel-system filled with 2 mL 37 °C pre-warmed Ringer’s solution. The artificial vessel system was fixed within a shaking device that was also pre-warmed to 37 °C. At certain time points, Ringer’s solution was withdrawn and the artificial vessel system was refilled with new warmed Ringer’s solution. Extinction of Ringer’s solution was measured and compared to that of reference Ringer’s solution. To determine hrAPC activity, 0.5 mg/mL of a chromogenic substrate of APC (Chromogenix S-2366, DiaPharma, West Chester Township, OH, USA) was mixed with 0.5 mg/mL pure APC (Haematologic Technologies Inc., Essex Junction, VT, USA) or with 10 mg/mL hrAPC (Xigris) dissolved in distillated water. Absorbance was measured with a photometer.

### Animal preparation

Ten male domestic pigs (weight 20–25 kg) were fasted overnight and then sedated with 12 mg/kg ketamine hydrochloride, 1 mg/kg xylazine, and 0.04 mg/kg atropine. Anesthesia was deepened with isoflurane and O_2_ via a mask. Intratracheal intubation was then performed to maintain anesthesia with 1.5–2.5 vol% isoflurane, 1.6–1.8 vol% O_2_, and 0.5 vol% N_2_O.

Arteriotomy of the right femoral artery was performed under sterile conditions. After administration of 100 IU/kg unfractionated heparin, a 6F introduction sheath was inserted. Heart rate, arterial blood pressure, electrocardiography, and temperature were monitored and arterial blood was sampled to control blood gases and acid–base balance throughout the procedure. Left and right coronary angiographies were performed using regular contrast medium (Ultravist, Bayer, Leverkusen, Germany).

Stents (BMSs and coated stents, 15 mm in length, 3 or 3.5 mm in diameter) were implanted either in the left anterior descending or left circumflex coronary arteries. Stent sizes and implantation pressures (8–12 atm, 30 s inflation) were chosen to achieve a stent:artery ratio of 1.1:1. A single stent was implanted in each artery. Pigs were randomized to receive either BMS or hrAPC-coated stents (up to two stents per animal).

After repeat coronary angiography, the guiding catheter and the introducer sheath were removed, the arteriotomy was ligated, and the skin was closed in two layers. Animals were allowed to recover from anesthesia and received metamizol for pain relief.

One day before coronary stent implantation, a loading dose of 250 mg aspirin and 300 mg clopidogrel was administered per os. During the 1-month follow-up, a daily dose of 100 mg aspirin and 75 mg clopidogrel was administered per os.

Animal investigations were carried out in accordance with the “Position of the American Heart Association on Research Animal Use,” as adopted by the AHA on November 11, 1984. The study was approved by the Ethics Committee on Animal Experimentation at the University of Kaposvar, Hungary.

### Control angiography after 4 weeks

At the 4-week follow-up, control angiography was performed under general anesthesia. After euthanasia with saturated potassium chloride, the heart was explanted and examined for evidence of infarction or fibrosis, especially in the location and perivascular region of the stents.

Coronary arteries were flushed with ~300 mL saline before pressure fixation in situ (100–110 mmHg) with 500 mL 4 % buffered formaldehyde. Coronary arteries were then dissected from the epicardial surface and fixed in 2 % buffered formalin for 24 h. Arteries with stents were embedded in Technovit 9100 (Heraeus Kulzer, Wehrheim, Germany). Hearts were then sectioned transaxially (along the short axis) at a minimum of 1-cm intervals. These sections underwent gross examination for evidence of myocardial infarction.

Serial peripheral venous blood samples were taken from the pigs before stenting as well as at 5 min, 10 min, 20 min, 30 min, and 1 day after stenting for measurements of APC (MyBioSource Inc., San Diego, CA, USA) and monocyte chemoattractant protein-1 (MCP-1; Abcam, Cambridge, UK). Samples obtained 10 min after implantation, at 1 week, and at 4 weeks were used to determine counts of eosinophils, platelets, and red and white blood cells.

### Quantitative assessment of coronary angiography

Pre-stent, post-stent, and follow-up quantitative angiographic parameters were measured with a computer-assisted quantitative coronary arteriographic edge-detection algorithm (ACOMPC, Siemens, Germany) by an investigator blinded to the stent type. In order to minimize variation in cardiac cycle-dependent dimensions, end-diastolic frames were chosen for the assessment of pre-, post-stent, and follow-up minimal lumen diameter, reference diameter, and percent diameter stenosis. Late lumen loss was calculated as the difference between post-stent and follow-up minimal lumen diameter.

### Histopathology and histomorphometry of stented arteries

Histological analyses were performed by an experienced investigator blinded to stent type [16, 17]. Five sections of the stented arteries were analyzed: the proximal reference segment (stent-free artery tissue within 10 mm from the stent edge); artery tissue at the proximal stent edge; artery tissue at the middle portion of the stent (stent body); artery tissue at the distal stent edge; and the distal reference segment (stent-free artery tissue within 10 mm from the stent edge). Arterial segments were stained with hematoxylin-eosin to determine the location and extent of injury.

The following histopathological parameters were measured: injury score, fibrin score, inflammation score, necrosis and hemorrhage scores, and endothelialization [16, 17]. Injury score was determined in accordance with Schwartz et al. [16]. Fibrin score was graded from 0 to 3 to reflect no fibrin deposition or mild, moderate, or heavy fibrin deposition involving <10 %, 10–25 %, or >25 % of the circumference of the vessel, respectively. Inflammation score was graded as 0 for no inflammation or <25 % struts with <10 inflammatory cells; 1 for up to 25 % struts with >10 inflammatory cells; 2 for 25–50 % struts with >10 inflammatory cells; 3 for >50 % struts with >10 inflammatory cells; and 4 for two or more struts-associated granulomatous inflammatory reactions. Necrosis and hemorrhage were scored as 0 if absent, 1 if mild, 2 if moderate, or 3 if heavy (if necrotic or hemorrhagic changes were seen around at least one stent strut). Endothelialization was evaluated as percent of stent struts covered by endothelium.

The following four quantitative histomorphometric parameters of the dilated segment, proximal reference segment, and distal reference segments were measured: lumen area; internal elastic-lamina area; external-elastic lamina area; and maximal neointimal thickness. Calculated histomorphometric parameters included: neointima area (difference between internal elastic-lamina and lumen areas); media area (difference between external-elastic lamina and internal elastic-lamina areas); percent area stenosis ((neointimal area/internal elastic − lamina area) × 100); remodeling index (external-elastic lamina area of the stented arterial segment/external-elastic lamina area of the proximal reference segment); and proximal or distal edge effects (neointimal area of the proximal or distal reference segment/neointimal area of the stented segment).

Myocardial sections beneath the stented arteries were dissected and stained with hematoxylin-eosin and evaluated for cellular infiltration, inflammation, and necrosis.

### P-selectin immunohistochemistry

Representative coronary-artery sections were stained with mouse anti-human P-selectin antibody (Genway Biotech, San Diego, CA, USA), which is known to cross-react with porcine tissue samples [18]. Sections were stained with hematoxylin-eosin for general morphology (Olympus Microscopy, Deutschland GmbH, Hamburg, Germany). The number of cells positive for P-selectin was calculated as the percentage of all visible cells.

### Statistical analysis

Continuous parameters are expressed as mean ± standard deviation. Categorical variables are reported as percentages. Continuous variables were compared with the two-tailed Student’s *t* test, while categorical variables were assessed with the Chi squared test. *P* values < 0.05 were considered significant. Statistical analyses were performed with SPSS version 17 for Macintosh.

## Results

### hrAPC coating and in vitro release kinetics

The control mechanism of the coating machine ensured that no residual drug was retained in the coating box. Each hrAPC-coated stent had a polymer-free coating of hrAPC with 2.3–2.6 µg hrAPC/mm^2^ of stent surface. Exposure to the chromogenic substrate indicated that the hrAPC coating was active, but also demonstrated hrAPC inactivation following repeated coating.

**Fig. 1:**
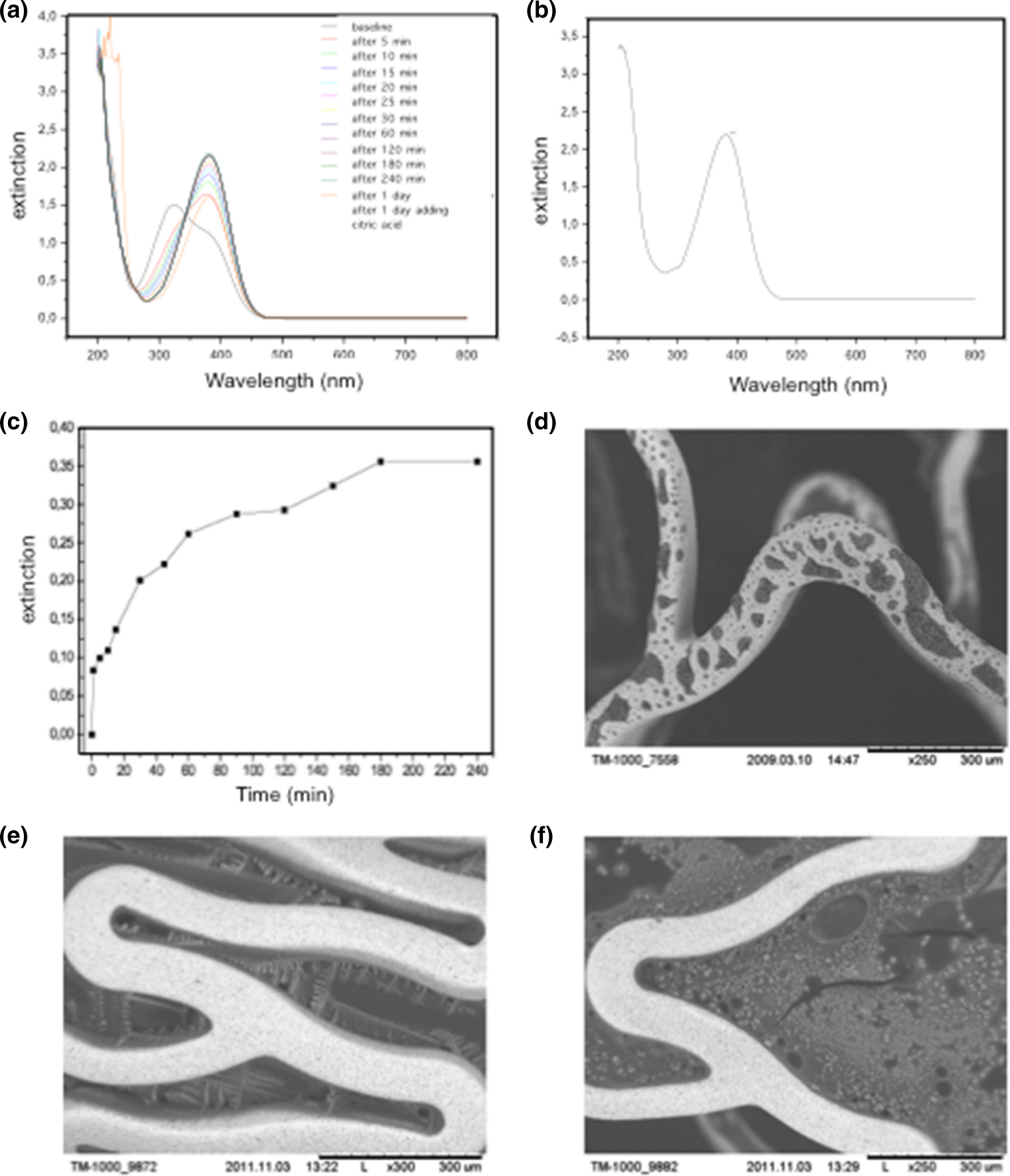
Coating of polymer-free stents with APC and hrAPC. **a**, **b** Extinction curves of chemical APC (**a**) and Xigris (*panel b*; containing 5 mg/mL hrAPC) mixed with Chromogenix S-2366. **c** Rapid release of hrAPC from the stent surface, with nearly complete release after 4 h. **d** Scanning electron microscopy of the hrAPC-coated expanded microporous stent. Note the uneven distribution of drug on the stent surface. **e** Scanning electron microscopy of the coated stent surface showing run-out of the drug components into the inter-strut space, with crystallization of the material on the stent-balloon surface. **f** Scanning electron microscopy of the stent and balloon surface after stent expansion

The in vitro drug-release curve revealed immediate release of the drug from the stent, with 50 % release at 30 min and nearly complete release after 4 h (Fig. 1a–c). Scanning electron microscopy showed complete coverage of the stent with hrAPC. However, the coating was not homogeneous (Fig. 1d), probably due to the presence of pharmaceutical substances used to maintain hrAPC as a dry powder. Additionally, run-out of the coating solution into the inter-strut spaces of the balloon surface occurred, likely due to incomplete adherence of the coating substance to the stent struts (Fig. 1e, f).

### APC and MCP-1 levels

**Fig. 2:**
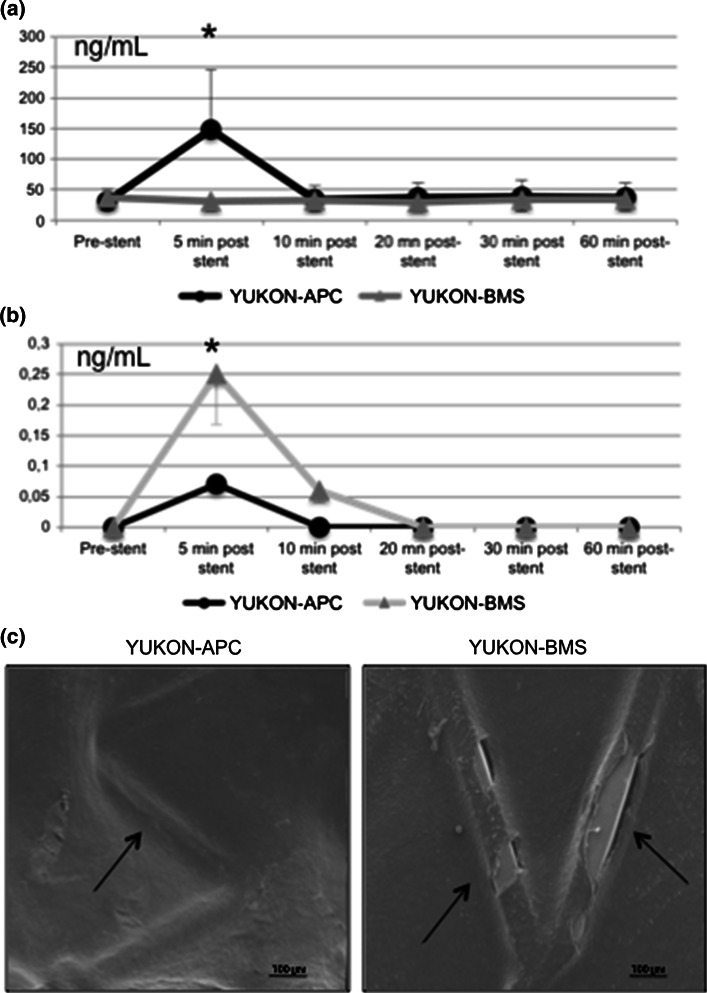
Time-dependent release of APC, changes in MCP-1 levels, and endothelialization of APC-coated stents and BMSs. **a** Release of APC immediately after stenting of porcine coronary arteries with hrAPC-coated stents (mean ± SD). **b** Differential increase in circulating MCP-1 levels after stent deployment (mean ± SD). **c** Scanning electron microscopy shows complete endothelialization of the hrAPC-coated stent (*left*, *arrow*) at 1-month follow-up, in contrast with the BMS (*right*, *arrows*)

APC levels were low prior to implantation, and immediately increased in pigs that received hrAPC-coated stents (Fig. 2a). However, 10 min later, APC levels dropped to baseline levels and remained low in these animals (Fig. 2a). MCP-1 was absent at baseline but increased slightly over time in both groups (Fig. 2b). This change was significant within minutes in animals with BMSs (Fig. 2b). MCP-1 levels returned to baseline after the first few minutes in both groups (Fig. 2b).

### Stenting procedural data

No procedural complications or allergic reactions were observed. Premature death, acute or sub-acute stent thrombosis, and concomitant disease were absent during follow-up. No changes in the numbers of red and white blood cells, platelets, or eosinophils were detected at any time point.

### Quantitative coronary angiography

Vessel diameters did not differ between stent groups before or after coronary procedures. At the 1-month follow-up, the mean minimal lumen diameter of animals with hrAPC-coated stents was significantly higher than that of animals with BMSs (2.51 ± 0.17 vs. 2.23 ± 0.32 mm, *P* = 0.033) and the percent area stenosis was smaller (16 ± 0.6 % vs. 26 ± 11 %, respectively, *P* = 0.041). Accordingly, late lumen loss was significantly smaller with hrAPC-coated stents than with BMSs (0.19 ± 0.17 vs. 0.47 ± 0.32 mm, *P* = 0.02).

### Histopathology

Histopathology indicated significantly less fibrin deposition and inflammation in the hrAPC-coated stent group than in the BMS group (Table 1). All animals had similar injury scores (Table 1). No granulomatous reaction or giant cells were documented. Myocardial histopathology uncovered no abnormalities. Scanning electron microscopy showed complete endothelialization of all hrAPC-coated stent struts, in contrast with incomplete endothelialization of stent struts in one BMS (Fig. 2c).

**Table 1:** Histopathologic results of the Yukon Choice bare metal stent (Yukon-BMS) and Yukon-APC (human recombinant activated protein C-coated stent)

	Yucon-APC (n = 10)	Yucon-BMS (n = 10)	*P* value
Injury score	1.35 ± 0.47	1.32 ± 0.52	0.821
Fibrin deposition score	0.55 ± 0.36	1.08 ± 0.32	**0.026**
Inflammation score	0.64 ± 0.24	0.98 ± 0.21	**0.021**
Haemorrhagia score	0	0.11 ± 0.08	0.556
Necrosis score	0	0	1
Endothelialization complete	100 %	90 %	0.136

Bold values indicate statistical significance (*p* < 0.05)

### Histomorphometric results

**Fig. 3:**
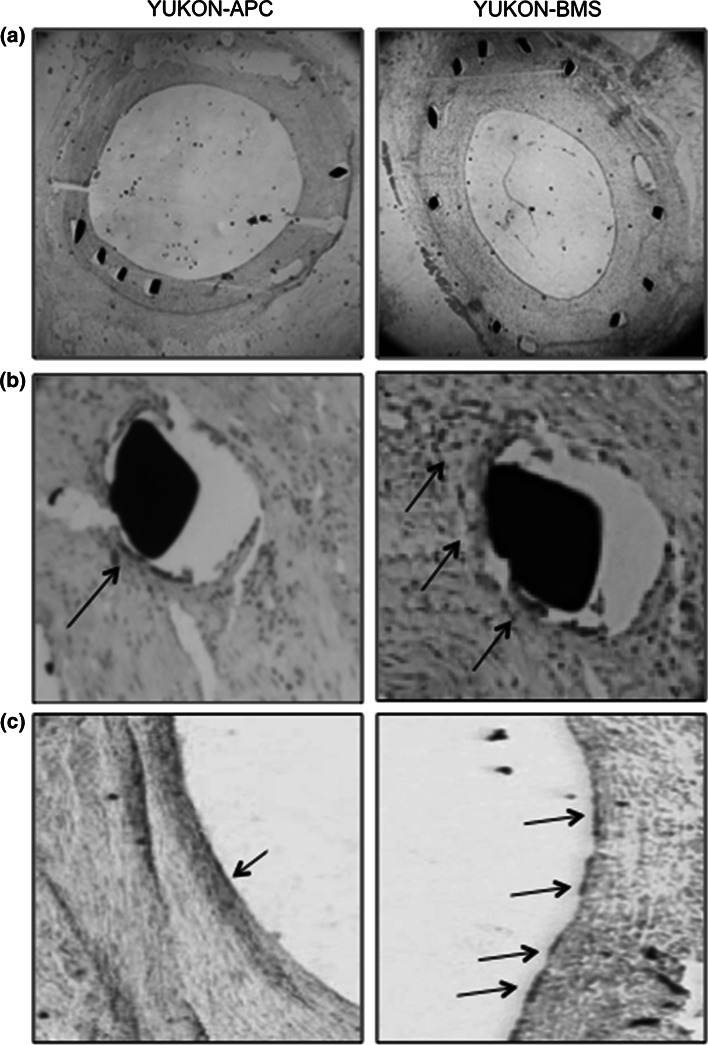
Histology (hematoxylin and eosin staining) and immunohistochemistry after implantation of hrAPC-coated or BMSs stents. **a** Histology of tissue 1 month after implantation with an hrAPC-coated stent (*left*) or a BMS (*right*). 2× magnification. Less neointimal hyperplasia occurred after implantation of the hrAPC-coated stent. **b** Less local inflammation occurred around the hrAPC-coated stent strut (*left*) than around the BMS strut (*right*). 40× magnification. **c** Immunohistochemistry of the porcine coronary artery with P-selectin antibody 1 month after implantation. Fewer P-selectin-positive endothelial cells (*arrows*) were detected in arteries implanted with hrAPC-coated stents (*left*) versus arteries implanted with BMSs (*right*)

Neointimal area, maximal neointimal thickness, and percent area of stenosis were significantly smaller with hrAPC-coated stents than with BMSs, with consequently higher lumen area in the hrAPC group (Table 2; Fig. 3). No vessel remodeling or edge effects were seen.

**Table 2:** Histomorphometric results of the Yukon Choice bare metal stent (Yukon-BMS) and Yukon-APC (human recombinant activated protein C-coated stent)

	Yucon-APC (n = 10)	Yucon-BMS (n = 10)	*P* value
Lumen area (mm^2^)	3.58 ± 0.87	3.11 ± 0.97	**0.025**
Neointimal area (mm^2^)	1.02 ± 0.92	1.57 ± 0.96	**0.013**
Internal elastic lamina area (mm^2^)	4.60 ± 0.44	4.68 ± 0.56	0.269
Media area (mm^2^)	0.68 ± 0.40	0.63 ± 0.34	0.304
External elastic lamina area (mm^2^)	5.28 ± 0.61	5.31 ± 0.59	0.424
Maximal neointimal thickness (mm)	0.26 ± 0.12	0.34 ± 0.11	**0.012**
% Area stenosis (%)	21.7 ± 18.3	33.5 ± 2.6	**0.011**
Remodeling index	0.88 ± 0.12	0.83 ± 0.15	0.778
Proximal edge effect	0.11 ± 0.07	0.12 ± 0.09	0.833
Distal edge effect	0.09 ± 0.1	0.12 ± 0.06	0.744

Bold values indicate statistical significance (*p* < 0.05)

Immunohistochemistry revealed significantly fewer P-selectin-positive endothelial cells in animals with hrAPC-coated stents than in animals with BMSs (4.6 ± 1.9 % vs. 11.6 ± 4.1 %, *P* < 0.001; Fig. 3).

## Discussion

Our results demonstrate the inhibitory effect of hrAPC-coated stents on neointimal hyperplasia, fibrin deposition, and vessel inflammation after implantation, even after short exposure of the arterial wall to hrAPC. Coating stents with hrAPC may therefore be a viable alternative to coating stents with antiproliferative drugs; APC is an endogenous protein that does not carry the risk of systemic or local toxicity. We successfully coated stents with Xigris, a drug approved by the FDA, but coating was not homogenous, probably due to additional pharmaceutical ingredients. However, this lack of homogeneity did not seem to negatively influence outcome.

### Possible beneficial effects of APC on neointimal hyperplasia

After stenting-induced vessel injury and compression of the atherosclerotic plaque, local platelet and fibrin deposition occur at the injury site, activating platelets and triggering interactions between leukocytes and platelets [19–21]. The subsequent inflammatory cascade leads to the discharge of growth factors and chemokines, which stimulate vascular smooth muscle cell proliferation and formation of extracellular matrix [22]. By inducing cell signaling, locally delivered APC may directly modulate this cellular response, exerting anti-inflammatory, cytoprotective, and barrier-protective activities [10]. APC has been shown to downregulate the expression of chemokines, MCP-1, and the intercellular adhesion molecule 1 family in human coronary artery endothelial cells [23]. APC was also effective against ischemia/reperfusion injury via its inhibition of endothelial apoptosis [24]. Use of APC was previously proposed for the management of microvascular inflammation [25]. Decreased levels of neointimal hyperplasia in animals implanted with hrAPC-coated stents in the present investigation support the hypothesis that APC, when coated on a stent, may exert beneficial effects on stenting-induced local inflammation. The short half-life of APC and its fast release kinetics are consistent with the observation that short exposure of the vessel wall to an antiproliferative substance inhibits neointimal proliferation [26, 27].

### APC against stent thrombosis

APC has the ability to protect the endothelial barrier [28], inhibiting the infiltration of inflammatory cells into adjacent tissues, interrupting the local inflammation cascade, and improving endothelial function [29]. These mechanisms may also underlie the quick endothelialization of stent struts observed here.

APC improves microvascular dysfunction [30] and decreases the expression and release of tissue factor on the surface of monocytes and on microparticles derived from activated platelets and endothelial cells [28, 30]. By inhibiting thrombin production, APC indirectly reduces platelet aggregation and degranulation as well as selectin-mediated adhesion of neutrophils to the endothelium [6]. In addition, in the early stages of local inflammation, P-selectin mediates the loose contact between leukocytes and platelets [19]. We detected significantly fewer P-selectin-expressing cells 1 month after implanting stents coated with hrAPC versus BMSs, which may reflect the inhibition of late thrombosis.

### Polymer-free coating of BMSs with hrAPC

The Translumina Stent Coating System enabled us to coat BMSs without polymer, with the inherent benefit of avoiding proinflammatory polymer carriers [31]. This equipment is suitable for coating stents with all ethanol-soluble substances, including antiproliferatives, angiotensin-converting enzyme inhibitors, and hormones. hrAPC is insoluble in absolute ethanol; therefore, we dissolved it in distilled water and dried it at room temperature. Although longer drying decreased the activity of hrAPC on the stent surface, our in vitro measurements of hrAPC release from the stent and our observation of increased levels of plasma APC demonstrate that our strategy resulted in stents coated with biologically sufficient amounts of hrAPC.

### Economic and clinical considerations of hrAPC-coated stents

We did not observe allergic reaction to hrAPC in the current investigation, probably due to the highly similar amino-acid sequences of porcine APC and hrAPC. Further, like endogenous APC, hrAPC is irreversibly inactivated by endogenous inhibitors in the blood, which form APC-inhibitor complexes that have no known biological activity [32]. hrAPC-coated stents contained very small amounts of drug, far below the regular dose of a single treatment in humans. Coating the stent with chemical APC (instead of Xigris) would likely result in more favorable outcomes, but coating a stent with a large molecule would require extensive manipulation of a biostable or biodegradable polymeric matrix in order to ensure constant and regulated drug release.

Despite the advantages of coating stents with hrAPC, the high costs of producing hrAPC may limit the widespread use of hrAPC-coated stents. However, economic studies of Xigris treatment for severe sepsis supported the cost effectiveness of this therapy [33]. Preventing restenosis and stent thrombosis using hrAPC-coated stents may be as cost effective as other drug-eluting stents. Although Xigris is no longer commercially available because it was withdrawn from the market in October 2011, hrAPC may still be an option as a coating for drug-eluting stents.

## Conclusion

In conclusion, coronary implantation of hrAPC-coated stents inhibited stent restenosis, development of neointimal hyperplasia, and vessel-wall inflammation in comparison with implantation of BMSs. Due to the antithrombotic, anti-inflammatory, antiapoptotic, and fibrinolytic properties of APC, coating stents with hrAPC may be a viable therapeutic option for preventing stent thrombosis and restenosis.
